# *Kakuna taibaiensis* sp. n. and a newly recorded species of *Dicranotropis* (Hemiptera, Fulgoroidea, Delphacidae) from China

**DOI:** 10.3897/zookeys.444.7810

**Published:** 2014-10-08

**Authors:** Feng-Juan Ren, Qi Xie, Li Qiao, Dao-zheng Qin

**Affiliations:** 1 Key Laboratory of Plant Protection Resources and Pest Management of the Ministry of Education, Entomological Museum, Northwest A&F University, Yangling, Shaanxi 712100, China; 2 Baoji University of Art and Science, Baoji, Shaanxi 721013, China; 3 Xinyang City Academy of Agricultural Sciences, Xinyang, Henan 464000, China

**Keywords:** Auchenorrhyncha, planthoppers, Fulgoromorpha, taxonomy, distribution

## Abstract

One new species of the delphacid genus *Kakuna* Matsumura, *Kakuna taibaiensis* Ren & Qin, **sp. n.** is described from Mt. Taibai in Shaanxi Province, China. *Dicranotropis montana* (Horvath, 1897) is reported for the first time from China. Habitus photos and illustrations of male genitalia of the two species are given.

## Introduction

The genus *Kakuna* was established by Matsumura (1935) for the type species *Kakuna kuwayamai* Matsumura, 1935 from Japan (Hokkaido, Sapporo). Ding (2006) regarded *Parametopina* Yang as a junior synonym of *Kakuna* Matsumura and transferred *Parametopina yushaniae* Yang to *Kakuna*. Recently, Chen and Yang (2010) redefined the generic characteristics and added three more species, *Kakuna lii*, *Kakuna nonspina* and *Kakuna zhongtuana* to the genus from southwestern China (Guizhou). To date, five *Kakuna* species are known all distributed in China and Japan. In this paper, we add a new species, *Kakuna taibaiensis* from Mt. Taibai (China: Shaanxi) and provide a key to all species in this genus.

Another delphacid species, *Dicranotropis montana*, was described by Horvath (1897), which was originally arranged as a member of the genus *Stiroma* Fieber. Asche (1982) studied the type material of this species and transferred it into *Dicranotropis* Fieber. This species is currently distributed in the Palaearctic Region. After checking the specimens collected in 2010 (now deposited in the Entomological Museum in NWAFU), we found this species in Hebei (northern China) and record it for the first time in the Chinese fauna.

## Material and methods

All specimens examined in this study are deposited in the Entomological Museum, Northwest A & F University, Yangling, Shaanxi, China (NWAFU). The genital segments of the examined specimens were macerated in 10% KOH and drawn from preparations in glycerin jelly with the aid of a light microscope. Illustrations of the specimens were made using a Leica MZ 12.5 stereomicroscope. Habitus photos were taken using a Scientific Digital micrography system equipped with an Auto-montage imaging system and a highly sensitive QIMAGING Retiga 4000R digital camera (CCD). Multiple photographs were compiled into final images. The terminology in this paper follows that of Ding (2006). Measurements of the body length were from the apex of the vertex to the posterior tip of the abdomen (macropterous) or to the tip of abdomen (brachypterous). All measurements are in millimeters (mm).

## Taxonomy

### Kakuna

Taxon classificationAnimaliaHemipteraDelphacidae

Matsumura, 1935

Kakuna Matsumura 1935: 76; Ding 2006: 404; Chen and Yang 2010: 30. Type species: *Kakuna kuwayamai* Matsumura, 1935, by original designation.
Parametopina Yang 1989: 308. Type species: *Parametopina yushaniae*Yang 1989: 308, synonymized by Ding 2006: 404.

#### Diagnosis.

Relatively large, brown delphacids. Head including eyes narrower than pronotum. Submedian carinae uniting at apex of vertex. Fastigium in lateral view rounded. Dorsum of body with a milky longitudinal stripe from middle of vertex to middle of posterior margin of forewing. Median carina of frons forked at extreme base. Antennae cylindrical. Forewing with large, longitudinal, brown marking. Metabasitarsus longer than tarsomere 2 + 3 combined, calcar thin, tectiform, with many black-tipped teeth on lateral margin. Male pygofer in lateral view with laterodorsal angle produced, medioventral process absent. Diaphragm of pygofer narrow, dorsal margin produced dorsad. Suspensorium ventrally ring-like. Aedeagus tubular, long. Parameres fairly developed, apically convergent. Anal segment deeply sunk into dorsal emargination of pygofer, ring-like, caudoventral processes present or absent.

#### Distribution.

China (Guizhou, Taiwan, Zhejiang, Fujian, Shaanxi), Japan.

#### Key to species in the genus *Kakuna* (males)

**Table:** 

1	Male anal segment produced caudoventrally	**2**
–	Male anal segment not produced caudoventrally	**3**
2	Male anal segment with two spine-like processes along caudoventral margin; mediodorsal processes of diaphragm separated at bases; aedeagus not bearing spinous processes at apex. China (Guizhou)	***Kakuna nonspinata* Chen & Yang**
–	Male anal segment with an elongate process at caudoventral margin; mediodorsal processes of diaphragm fused at their bases; aedeagus bearing spinous processes at apex. China (Taiwan)	***Kakuna yushaniae* (Yang)**
3	Aedeagus in profile distinctly expanded at apex, dorsal margin with spinous processes	**4**
–	Aedeagus in profile not expanded at apex, dorsal margin without spinous processes	**5**
4	Mediodorsal processes of diaphragm curved laterad apically; aedeagus without spinous processes ventrally near apex. China (Guizhou)	***Kakuna lii* Chen & Yang**
–	Mediodorsal processes of diaphragm straight apically; aedeagus with spinous processes ventrally near apex. China (Zhejiang, Fujian, Guizhou), Japan (Hokkaido, Honshu, Kyushu)	***Kakuna kuwayamai* Matsumura**
5	Mediodorsal processes of diaphragm long, reaching to the level of anal segment; aedeagus in profile curved ventrad medially; inner margins of parameres with denticles medially. China (Shaanxi)	***Kakuna taibaiensis* Ren & Qin, sp. n.**
–	Mediodorsal processes of diaphragm short, not reaching to the level of anal segment; aedeagus in profile curved dorsad medially; inner margins of parameres without denticles but with a nipple-like process medially. China (Guizhou)	***Kakuna zhongtuana* Chen & Yang**

### Kakuna taibaiensis

Taxon classificationAnimaliaHemipteraDelphacidae

Ren & Qin sp. n.

http://zoobank.org/0DE83AE8-F62C-4649-8C6E-459D960AA940

Figs 1 –16

#### Description.

Macropterous male: Body length: male 5.82–5.91 mm; forewing length: male 5.06–5.13 mm (n=2).

*Color.* General color brown. Ocelli reddish brown, eyes black. Dorsum of body with a milky longitudinal stripe from the junction of Y-shaped carina to the middle of posterior margin of forewing. Forewing yellowish brown, membrane has a large, longitudinal, fuscous marking from base of costal area to apex, veins fuscous, longitudinal veins ornamented with blackish brown granules. Abdomen fuscous. Fore and middle legs brown, hind legs yellowish brown, apices of spines on tibiae and tarsi black.

**Figures 1–5.**
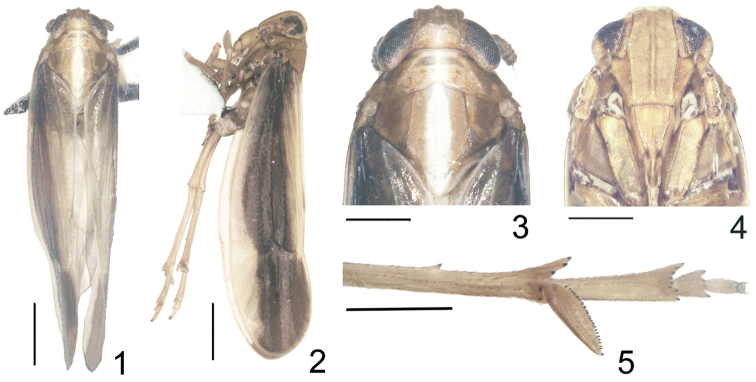
*Kakuna taibaiensis* Ren & Qin, sp. n. **1** male adult, dorsal view **2** male adult, left lateral view **3** head and thorax, dorsal view **4** frons and clypeus **5** metatarsus and post-tibial spur. Scale bars = 1.0 mm (Figs **1, 2**); 0.5 mm (Figs **3–5**).

*Structure.* Head including eyes narrower than pronotum (about 0.81:1) (Figs 1, 3). Vertex shorter in midline than wide at base (about 0.82:1), narrower at apex than at base (about 0.78:1), lateral margins slightly concave in dorsal view, submedian carinae convex, originating from near 1/3 base of lateral carinae and uniting at apex of vertex (Figs 1, 3). Y-shaped carina distinct, basal compartment shallowly concave (Fig. 3). Fastigium rounded (Fig. 2). Frons longer in midline than maximum width about 2.05:1, widest above the level of ocelli, median carina conspicuous, forked at extreme base (Fig. 4). Postclypeus wider at base than frons at apex, post- and anteclypeus together approximately 0.86 × of the length of frons (Fig. 4). Rostrum almost reaching mesotrochanters. Antennae terete, nearly attaining middle level of postclypeus, scape longer than wide at apex (about 1.83:1), shorter than pedicle (about 0.52:1) (Fig. 4).

Pronotum in midline slightly shorter than length of vertex (about 0.79:1), lateral carinae developed, slightly curved, not reaching posterior margin (Fig. 3). Mesonotum medially ca. 1.64 times longer than vertex and pronotum together, lateral carina almost straight, reaching posterior margin, median carina obscure apically (Fig. 3). Forewings long and narrow, longer than widest part about 3.35:1, widest in middle (Figs 1, 2, 16). Spination of apex of hind leg 5 (3+2) (tibia), 8 (6+2) (basitarsus) and 4 (2nd tarsomere) (Fig. 5). Metabasitarsus distinctly longer than tarsomere 2+3 combined (about 1.79:1), calcar shorter than metabasitarsus (about 0.77:1), thin, bearing 29 black-tipped teeth on lateral margin (Fig. 5).

**Figures 6–16.**
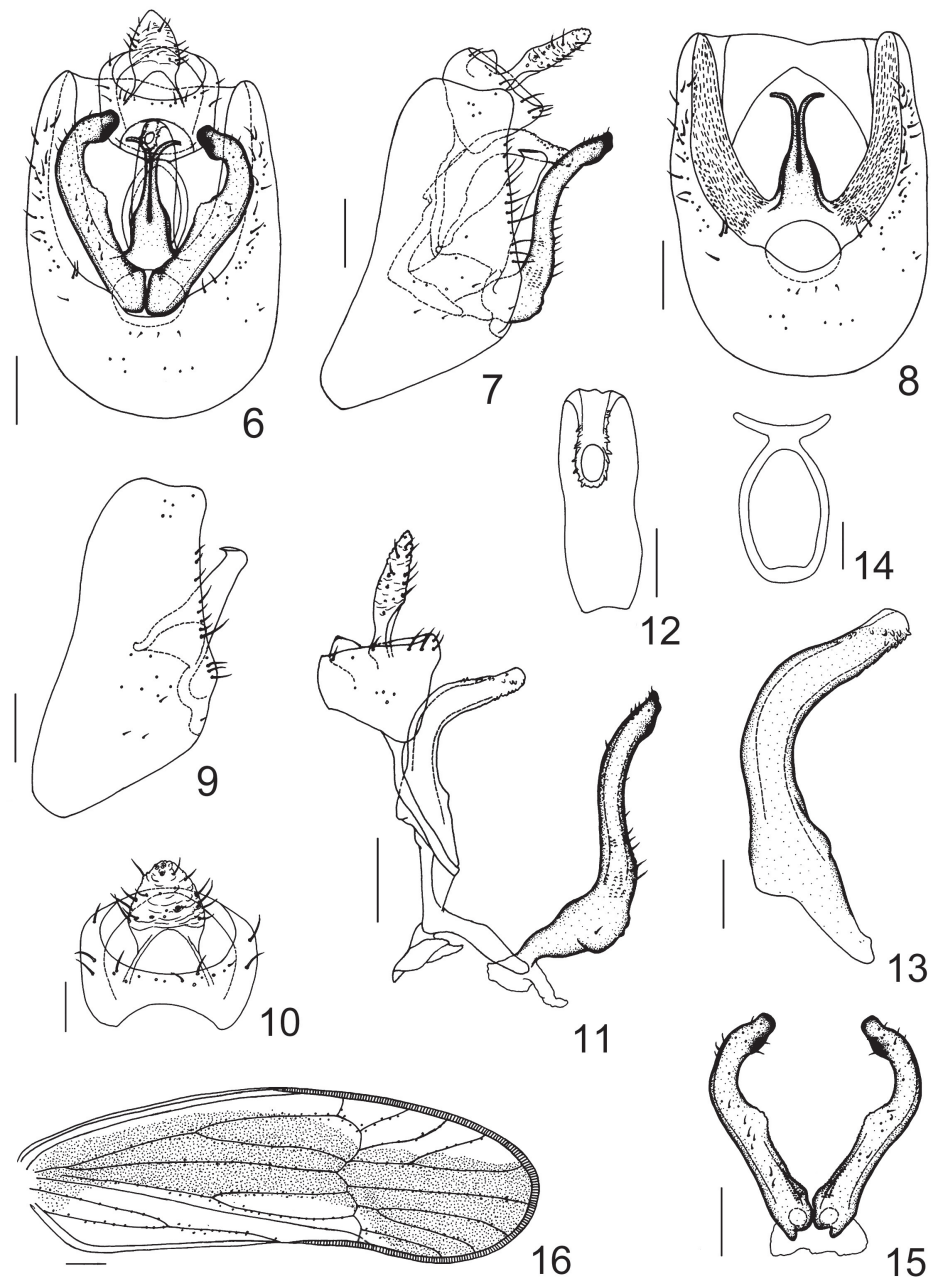
*Kakuna taibaiensis* Ren & Qin, sp. n. **6** male genitalia, posterior view **7** male genitalia, left lateral view **8** male pygofer, posterior view **9** male pygofer, left lateral view **10** anal segment, posterior view **11** anal segment, aedeagal complex, connective and parameres, left lateral view **12** aedeagus, dorsocaudal view **13** aedeagus, left lateral view **14** suspensorium, posterior view **15** parameres, posterior view **16** forewing. Scale bars = 0.2 mm (Figs **6–9, 11, 15**); 0.1 mm (Figs **10, 12–14** ); 0.5 mm (Fig. **16**).

*Male genitalia.* Male pygofer slightly wider ventrally than dorsally, laterodorsal angles roundly produced caudad; in posterior view with opening longer than wide, ventral margin shallowly excavated, medioventral process absent (Figs 6–9). Suspensorium ventrally ring-like, dorsally with a process at each side leading to the anal segment ventrolaterally (Fig. 14). Diaphragm narrow, mediodorsal processes fairly developed, pillar-like, basally with a broad common stalk, thence contiguous apicad, apical part separated and curved laterad, tips truncated (Figs 6, 8). Parameres fairly long, reaching to the level of anal segment, in posterior view contiguous basally, apical 2/5 convergent mesad, apices rounded, inner margins expanded and ornamented with denticles medially (Figs 6, 11, 15). Aedeagus tubular, arch-shaped in profile, moderately dilated near the base, near apex on the dorsal side to the ventral apex provided with small teeth, gonopore apical on the slightly membranous dorsal side (Figs 12, 13). Male anal segment deeply sunk into dorsal emargination of pygofer, ring-like, caudoventral processes absent (Figs 6, 7, 10, 11).

#### Type materials.

**Holotype.** ♂ (macropterous, NWAFU), China, Shaanxi Province, Mt. Taibai, 13-VIII-2010, by light trap, coll. A. P. Dong. **Paratype.** 1♂ (macropterous, NWAFU), same data as holotype.

#### Female.

Unknown.

#### Host plant.

Unknown.

#### Etymology.

The species epithet is named after the type locality, Mt. Taibai in Shaanxi, China.

#### Distribution.

Known currently from the type locality in northwest China (Shaanxi Province).

#### Remarks.

*Kakuna taibaiensis* is similar to *Kakuna zhongtuana* Chen & Yang (2010) in the male anal segment not produced caudoventrally, aedeagus not bearing spinous processes and mediodorsal processes of diaphragm having a common stalk basally. However, the new species differs from the latter in the mediodorsal processes fairly long, reaching to the level of anal segment (mediodorsal processes short, not reaching to the level of anal segment in *zhongtuana*), aedeagus curved ventrad medially in profile (aedeagus curved dorsad medially in profile in *zhongtuana*), parameres rounded at apex in posterior view, inner margins ornamented with denticles medially (parameres acute at apex and adorned with a nipple-like process medially along each inner margin in *zhongtuana*).

#### Discussion.

The Himalaya-Qinling-Huai River line is the most distinctive barrier and may serve as the division of the Palaearctic and Oriental Regions since the Pleistocene. However, the north-south transitional affects have been much more pronounced in species and a broad transitional zone has resulted (Zhang 2002). The new finding in this paper based on the specimens from Mt. Taibai (the main peak of Mts. Qinling in Shaanxi, China) confirms the suggestion of Chen and Yang (2010) that the members of the genus *Kakuna* have extended into the southern area of the Palaearctic Region. During our investigations of Delphacidae on Mt. Taibai, we found many species in this family have extended into the border of the two Regions which were traditionally thought to be confined in the Palaearctic or Oriental Region only, including some new species described in recent years (Qin 2007, Qin et al. 2012). We suspect that the delphacid fauna in this border area will be more extensive if more investigations are conducted.

### Dicranotropis montana

Taxon classificationAnimaliaHemipteraDelphacidae

(Horvath, 1897) new record to China

Figs 17 –34

Stiroma montana Horvath 1897: 625.
Dicranotropis tenellula Dlabola 1965: 84; Emeljanov 1977: 109, synonymized by Asche 1982: 30.
Dicranotropis gratiosa Dlabola 1997: 315, synonymized by Holzinger 1999: 259.
Dicranotropis montanus Vilbaste 1965: 15, synonymized by Asche 1982: 30.
Dicranotropis montana (Horvath), Asche 1982: 30; Holzinger 1999: 259; Holzinger et al. 2003: 267; Nickel 2003: 55; Trivellone 2010: 100.

#### Description.

Macropterous male: Body length (from apex of vertex to the tip of forewing): male 3.40–3.64 mm, female 3.44–3.90 mm; forewing length: male 2.72–2.96 mm, female 3.04–3.24 mm. Brachypterous male: Body length (from apex of vertex to the tip of abdomen): male 2.24–2.56 mm, female 2.64–2.96 mm; forewing length: male 0.99–1.08 mm, female 1.04–1.24 mm.

*Color.* General color of male (macropterous) blackish brown. Ocelli reddish black, eyes grayish black. Vertex anteriorly, frons, clypeus, lateral area of pronotum behind eyes black. Antennae yellowish brown except apex of scape and base of pedicle fuscous. Pronotum between lateral carinae and laterobasal angles sordid whitish. All carinae and margins of vertex, frons and clypeus whitish. Rostrum fuscous at apex. Mesonotum mostly dark brown, scutellum whitish apically. Abdomen dark. Legs brown to yellowish brown. Tegmina subhyaline, veins yellowish brown. Female with ovipositor brown. Male (brachypterous) with the same color as macropterous except pronotum, mesonotum and tegmina yellowish brown, abdomen of female mostly yellowish white, abdomen with small brown spots dorsally and ventrally on each segment.

**Figures 17–22.**
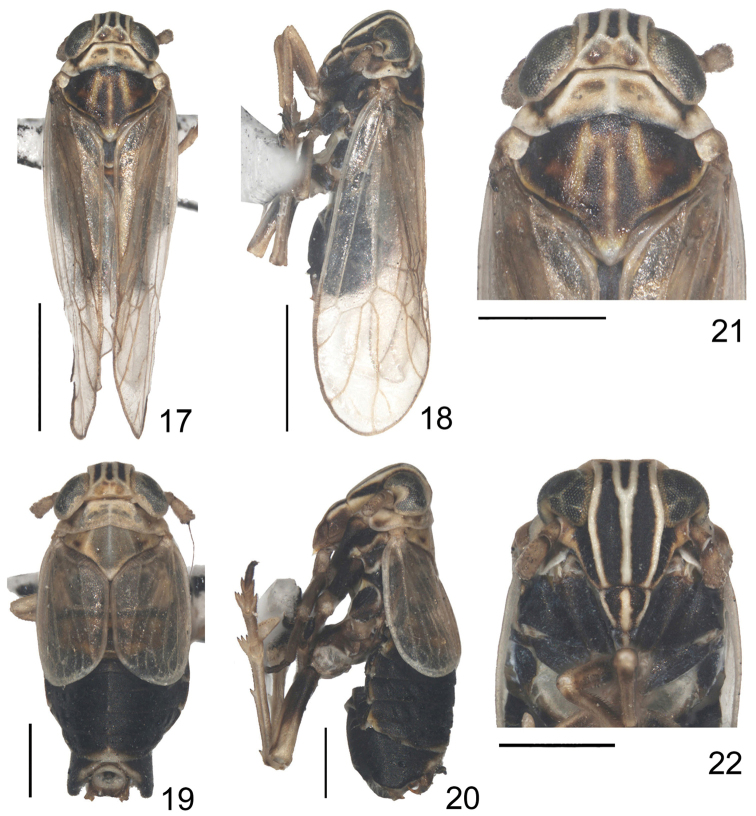
*Dicranotropis montana* (Horvath, 1897) **17** macropterous male, dorsal view **18** macropterous male, left lateral view **19** brachypterous male, dorsal view **20** brachypterous male, left lateral view **21** head and thorax (macropterous male), dorsal view **22** frons and clypeus (brachypterous male). Scale bars = 1.0 mm (Figs **17, 18**); 0.5 mm (Figs **19–22**).

*Structure.* Head including eyes slightly narrower than pronotum (about 0.92:1). Vertex shorter in midline than wide at base (about 0.62:1), narrower at apex than at base (about 0.89:1). Submedian carinae originating from near 1/4 base of lateral carinae, not uniting at apex of vertex. Y-shaped carina distinct (Figs 17, 19, 21). Frons longer in midline than maximum width about 1.64:1, widest above the level of ocelli, carinae conspicuous, median carina forked at basal fourth (Fig. 22). Postclypeus broader at base than frons at apex, postclypeus and anteclypeus together approximately 0.80 × the length of the frons (Fig. 22). Rostrum almost reaching mesotrochanters. Antennae terete, reaching frontoclypeal suture, scape longer than apical width (about 1.59:1), shorter than pedicle (about 0.64:1) (Figs 18, 20, 22).

Pronotum shorter than vertex in midline (about 0.91:1), lateral carinae straight, not reaching to posterior margin (Figs 17, 19, 21). Mesonotum medially ca. 1.34 times longer than vertex and pronotum together, lateral carina reaching posterior margin, median carina obscure apically (Figs 17, 21). Macropterous forewings surpassing tip of abdomen approximately 2/5 of its total length (Figs 17, 18), longer than widest part (about 2.86:1). Spinal formula of hind leg 5–7–4, post-tibial spur shorter than metabasitarsus, sparsely with about 8 tiny teeth along hind margin.

**Figures 23–34.**
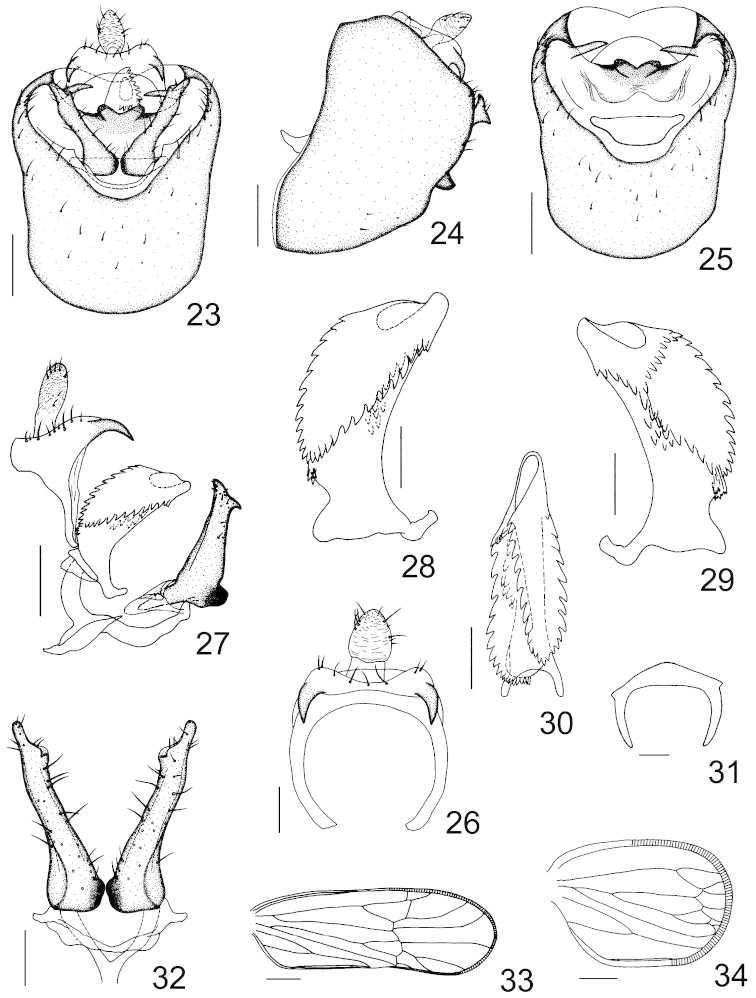
*Dicranotropis montana* (Horvath, 1897). **23** male genitalia, posterior view **24** male genitalia, left lateral view **25** male pygofer, posterior view **26** anal segment, posterior view **27** anal segment, aedeagal complex, connective and parameres, left lateral view **28** aedeagus, left lateral view **29** aedeagus, right lateral view **30** aedeagus, dorsal view **31** suspensorium, posterior view **32** parameres, posterior view **33** macropterous forewing **34** brachypterous forewing. Scale bars = 0.2 mm (Figs **23–25, 27**); 0.1 mm (Figs **26, 28–30, 32**); 0.04 mm (Fig. **31**); 0.5 mm (Fig. **33**); 0.25 mm (Fig. **34**).

*Male genitalia.* Male pygofer in profile wider ventrally than dorsally, anterior margin distinctly convex submedially (Fig. 24); in posterior view opening of pygofer obcordate, medioventral process absent (Figs 23, 25), below laterodorsal angle interiorly with a spine-like process on each side, transverse-directed (Figs 23, 25). Suspensorium n-shaped, dorsally arched medially with two small triangular processes on both ends (Fig. 31). Diaphragm broad, mediodorsal processes strongly sclerotized and laterally beset with many granulations, incised medially (Figs 23, 25). Opening for parameres large, dorsal margin nearly straight, lateral margins slightly sinuate (Fig. 25). Parameres long, contiguous at bases, narrowed and divergent apically, inner margins expanded subapically, in profile apical margin emarginated in two triangular processes (Fig. 27). Aedeagus tubular, short and broad, with five rows of teeth on surface, including four longitudinal rows and one transverse row basad of gonopore (Figs 28, 29, 30). Male anal segment ring-like, laterodistal angles produced into a short, stout spinose process on each side (Figs 23, 24, 26, 27).

#### Species examined.

23 ♂♂ 22 ♀♀ (macropterous) and 35 ♂♂ 46 ♀♀ (brachypterous), China: Hebei Province, Mt. Xiaowutai, 24-VI-2009, coll. D. Z. Qin.

#### Distribution.

China (Hebei), Russia, Austria, Switzerland, Germany, France, Italy, Hungary, Romania, Mongolia.

#### Host plant.

Unknown.

#### Discussion.

Dlabola (1965) established *Dicranotropis tenellula* Dlabola based on the specimens from Mongolia; Vilbaste (1965) described *Dicranotropis montana* Vilbaste from Russia but it was regarded as a junior synonymy of *Dicranotropis tenellula* Dlabola by Emeljanov (1977). Asche (1982) treated both *Dicranotropis tenellula* Dlabola and *Dicranotropis montana* Vilbaste as junior synonyms of *Dicranotropis montana* (Horvath, 1897). However, the treatment of *Dicranotropis tenellula* Dlabola was not accepted by Anufriev and Emeljanov (1988). Holzinger et al. (2003) studied the *Dicranotropis* species in central Europe, in *Dicranotropis montana* (Horvath) part, they redrew the male genitalia of this species and noted: “according to Emeljanov and Gnezdilov (pers. common.), the central Asian *Dicranotropis tenellula* Dlabola, 1965 is not conspecific with *Dicranotropis montana* (Horvath, 1897)”. After checking the specimens deposited in NWAFU, and also these illustrations of male genitalia provided by Dlabola (1965, 1997), Vilbaste (1965), Anufriev and Emeljanov (1988), Holzinger (1999) and Holzinger et al. (2003), we found *Dicranotropis tenellula* Dlabola to be very similar to *Dicranotropis montana* (Horvath) and very difficult to distinguish. We hope the status of *Dicranotropis tenellula* can be reconsidered and firmly established using more advanced methods in the future. Here we consider *Dicranotropis tenellula* Dlabola as a junior synonym of *Dicranotropis montana* (Horvath).

## Supplementary Material

XML Treatment for Kakuna

XML Treatment for Kakuna taibaiensis

XML Treatment for Dicranotropis montana
